# Mandibular first and second molars with three mesial canals: a case series 

**Published:** 2010-02-20

**Authors:** Mohsen Aminsobhani, Behnam Bolhari, Noushin Shokouhinejad, Abdollah Ghorbanzadeh, Sholeh Ghabraei, Mohamad Bagher Rahmani

**Affiliations:** 1 *Department of Endodontics, Dental School/Dental Research Center, Tehran University of Medical Sciences, and Iranian Center for Endodontic Research, Tehran, Iran*; 2 *Department of Endodontics, Dental School/Dental Research Center, Tehran University of Medical Sciences, Tehran, Iran*; 3 *Dentist, Private Practice*

**Keywords:** Four canals tooth, Mandible, Molar, Root canal anatomy

## Abstract

Adequate cleaning, shaping and filling of the root canal system are mandatory for successful root canal treatment. Thorough knowledge of root canal morphology and unusual anatomy of the tooth is critical for the practitioner. The occurrence and location of the third mesial canal (Middle Mesial Canal) in mandibular first and second molars in relation to other two mesial canals that were treated in private practice were studied. In 27 clinical cases, the presence of a middle mesial canal was demonstrated. The third canal was located in the middle of the distance between the mesiobuccal and mesiolingual canals. This canal configuration was found in six second lower molars and twenty one first molars. Middle mesial canal in all of our cases joined to mesiobuccal or mesiolingual canals. None of the teeth consisted of three independent canals with three apical foramina. In conclusion, every attempt should be made to find and treat all root canals of a tooth.

## Introduction

Successful root canal treatment depends on adequate cleaning, shaping, and filling of the root canal system. It is critical that the practitioner has thorough knowledge of root canal morphology of each individual tooth. 

The two-rooted mandibular first permanent molars usually have three canals. Two root canals are located in mesial root and another one in distal root. Hess reported that the prevalence of three root canals in mandibular molars was 78% (1). *In vitro* studies by Skidmore and Bjorndal (2) demonstrated the prevalence of two root canals in distal root of mandibular molars was almost 30%.

However, unusual root canal anatomy associated with the mandibular first molars has been reported in several studies and case reports (3-10). Vertucci and William (11), as well as Barker *et al.* (12) described the presence of a middle mesial canal (MMC). Middle mesial canal is sometimes present in the developmental groove between MB and ML canals (8). In a radiographic study of extracted teeth Goel *et al.* (5) reported that mandibular first molars had three mesial canals in 13.3%, four mesial canals in 3.3%, and three distal canals in 1.7% of specimens. In a clinical study of 145 mandibular first molars, Fabra-Campos found four molars (2.07%) with five canals- three in mesial root and two in distal (4). In the four cases, the MMC did not show an independent apical foramen. On the other hand, it has been reported very few mandibular first and second molars with three separated canals in mesial root (3,6,7,13-16). Shahi *et al.* evaluated the distribution of the root canal number and configuration in the 209 mandibular first permanent molars in Iranian population and found that 0.95% of the mesial roots had three mesial canals but they did not show canal configuration of this root canals (17).

**Figure 1:**
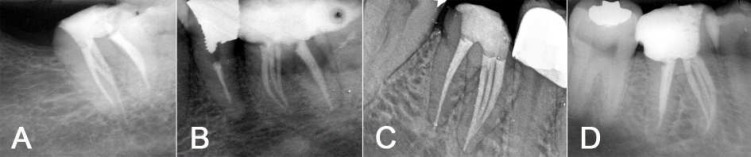
A) Mandibular second molar (2-3-2), B) mandibular first molar (3-2), C) mandibular first molar (3-1), D) mandibular first molar (3-2-1)

They mentioned that three canals with separate foramina were not seen in any of the roots studied. In Ahmed *et al.*’s study, in a Sudanese population using a clearing technique, the prevalence of three mesial canals was 4% in mandibular first molars and 10% in mandibular second molars (18).

In a clinical study of 251 root-canal treated permanent mandibular first molars in a Saudi Arabian sub-population, the teeth were examined clinically and radiographically and all mesial roots had two root canals and no unusual canal configuration was observed (19). Reuben *et al.* evaluated root canal morphology of 125 extracted mandibular first molars in an Indian population by using spiral computed tomography (SCT); they did not find mandibular molar mesial roots with three mesial canals (20).

In the current study, 27 cases of mandibular first and second molars with three mesial canals and one or two distal canals in Iranian population were reported. 

## Case Series

A total of 27 mandibular molars with three mesial canals were treated from 2000 to 2009 in our practice. Twenty two cases received initial root canal therapy (RCT); and in five cases, endodontic re-treatment was performed. Two were treated by a general practitioner and the rest were treated by endodontists in their private practice.

Of the 27 mandibular molars, 21 teeth (77.8%) were first molars and 6 teeth (22.2%) were second molars. Two cases showed 2 orifices, 3 root canals, and 2 apical foramina "2-3-2" (Figure 1A). Three orifices and 2 apical foramina "3-2" were seen in 14 cases (Figure 1B); three orifices and one apical foramen "3-1" in 4 cases (Figure 1C); and 3 orifices, 2 root canals, and 1 apical foramen "3-2-1" were seen in 7 cases (Figure 1D). The variations of canal configurations of mesial roots of the treated teeth are shown in Table 1. 

Of the 14 cases which showed type "3-2" root canal morphology, in 10 cases the middle mesial canal joined the mesiobuccal canal at the apical area; and in 4 cases, MMC and mesiolingual canals joined together in the apical third of the mesial root. In all of 7 cases that showed type "3-2-1" root canal morphology, the MMC joined the mesiobuccal canal to make a single canal. Subsequently, this canal joined the mesiolingual canal ending in one apical foramen at the apical area of the mesial root. 

## Discussion

Before root canal treatment is performed, the clinician should ideally have adequate knowledge of the pulp chamber and internal anatomy of the teeth. All root canals should be accessed, cleaned, and shaped to achieve a hermetic obturation of the entire root canal space. 

There is an abundant amount of reports that relate the anatomic variations of mandibular molars (1). This should induce the clinician to accurately observe the pulp chamber floor to locate possible canal orifices. This will increase the long term prognosis of endodontic therapy. Searching for additional canal orifices should be standard practice for clinicians. A round bur or an ultrasonic tip can be used for removal of any protuberance from the mesial axial wall which would prevent direct access to the developmental groove between MB and ML orifices. This developmental groove should be carefully checked with the sharp tip of an endodontic explorer. If depression or orifices are located, the groove can be troughed with ultrasonic tips at its mesial aspect until a small file can negotiate this intermediate canal (8). 

**Table 1:** Mesial root canal pattern (Vertucci)

Pattern	2-3-2	3-2	3-1	3-2-1
Tooth				
Mandibular 1^st^ molar	**0**	**13**	**4**	**4**
Mandibular 2^nd^ molar	**2**	**1**	**0**	**3**
Total	**2**	**14**	**4**	**7**

New technologies, such as the dental operating microscope and dental loupes, offer magnify-cation and illumination of the operating field and substantially improve the visualization of root canal orifices (21,22). We did not use magnification or these new technologies during treatment sessions (Figure 5). It is possible that more cases may have been discovered with magnification and extra illumination.

Numerous studies in the past decades have described the morphology of teeth including mandibular molars (23). The morphology of the mesial root canals in mandibular molars is complex and difficult to find, with a high frequency of inter-canal communications and or isthmuses (2,5,18,23-26). 

The presence of a third canal (middle mesial) in the mesial root of the mandibular molars has been reported to have an incidence of 0.95%-15% (4,8,9,12,17,23,24,27,28). In almost all of the clinical cases reported until today, this canal joined the mesiobuccal or mesiolingual canal in the apical third (3,4,7,29,30). However a few mandibular first molars that had three independent canals in their mesial root have been reported (3,6,7,13-16). In this case seriess no mesial root with three distinct and independent canals was discovered which concurrs with other studies (6,17,27).

In 12 cases (44.5%), MMC joined the mesiobuccal canal in the apical third. In 4 cases (14.8%), it joined to the mesiolingual canal in the apical area. These finding are in agreement with Fabra-Campos (4) who showed that MMC joined to mesiobuccal canal in most cases. 

Most of the reported and reviewed cases in literature look at first mandibular molars and a few clinical cases that have been reported are second mandibular molars. In our case series, of the 27 molars treated, 21 (77.8%) were first molar and the others were second molar. This correlates with Barker's findings (12).

## Conclusion

In conclusion, every attempt should be made to find and treat all root canals to ensure successful endodontic treatment. The importance of an accurate clinical evaluation of root canal number and morphology in mandibular molars cannot be overemphasized.
